# Supplementary material for: Multiple alleles at a single locus control seed dormancy in Swedish Arabidopsis
Source: eLife. 2016 Dec 14;5:e22502. doi: 10.7554/eLife.22502 (PMC5226650; doi:10.7554/eLife.22502)
Supplement: Figure 3—source data 6. — The underlined sequences correspond to the flanking sequences of the EcoRI restriction site in the pGreenII 0029 that was used for transformation. DOI: http://dx.doi.org/10.7554/eLife.22502.014 [file elife-22502-fig3-data6.docx]

**Figure 3-source data 6. *DOG1* PCR primers used in the complementation experiment.** We designed an Eden-2 specific forward primer because of structural variants at the 3’ end of *DOG1*. The underlined sequences correspond to the flanking sequences of the EcoRI restriction site in the pGreenII 0029 that was used for transformation.

| **ID** | **Name** | **Forward** | **Reverse** |
| --- | --- | --- | --- |
| 992 | Ale-Stenar-44-4 | CGGGCTGCAGGAATTGGGGCATCTAGAATTGTGTCAT | GCTTGATATCGAATTTGACTTCGGCTTCTTTTTAACC |
| 1002 | Ale-Stenar-64-24 | CGGGCTGCAGGAATTGGGGCATCTAGAATTGTGTCAT | GCTTGATATCGAATTTGACTTCGGCTTCTTTTTAACC |
| 6913 | Eden-2 | CGGGCTGCAGGAATTGCTTACTAATCGCGATCCAAAC | GCTTGATATCGAATTTGACTTCGGCTTCTTTTTAACC |
