# Supplementary material for: Multiple alleles at a single locus control seed dormancy in Swedish Arabidopsis
Source: eLife. 2016 Dec 14;5:e22502. doi: 10.7554/eLife.22502 (PMC5226650; doi:10.7554/eLife.22502)
Supplement: Supplementary file 1. — Partial Mantel correlations between climate variables and GR21, where the kinship between accessions is included as a covariate in the model to correct confounding caused by population structure. The Mantel r values are the Spearman partial correlation coefﬁcients. DOI: http://dx.doi.org/10.7554/eLife.22502.018 [file elife-22502-supp1.docx]

**Supplementary file 1. Correlation between GR21 and climate variables.** Partial Mantel correlations between climate variables and GR21, where the kinship between accessions is included as a covariate in the model to correct confounding caused by population structure. The Mantel *r* values are the Spearman partial correlation coefﬁcients.

| **Climate variable** | **Mantel *r*** | ***P* value** |
| --- | --- | --- |
| Mean Temperature of Driest Quarter | 0.27 | 0.001 |
| Annual Mean Temperature | 0.24 | 0.001 |
| Length of the growing season | 0.24 | 0.001 |
| Mean Temperature of Coldest Quarter | 0.23 | 0.001 |
| Temperature Seasonality | 0.19 | 0.001 |
| Mean Temperature of Warmest Quarter | 0.17 | 0.001 |
| Mean Temperature of Wettest Quarter | 0.14 | 0.001 |
| Precipitation Seasonality | 0.10 | 0.001 |
| Number of consecutive frost-free days | 0.10 | 0.001 |
| Aridity | 0.10 | 0.001 |
| Mean daylength | 0.09 | 0.001 |
| Number of consecutive cold days | 0.09 | 0.001 |
| Isothermality | 0.06 | 0.001 |
| Annual photosynthetically active radiation | 0.05 | 0.003 |
| Mean relative humidity | 0.04 | 0.007 |
| Precipitation of Wettest Quarter | -0.00 | 0.988 |
| Precipitation of Coldest Quarter | -0.00 | 0.633 |
| Precipitation of Driest Quarter | -0.03 | 0.022 |
| Precipitation of Warmest Quarter | -0.03 | 0.025 |
| Annual Precipitation | -0.03 | 0.014 |
