# Supplementary material for: Multiple alleles at a single locus control seed dormancy in Swedish Arabidopsis
Source: eLife. 2016 Dec 14;5:e22502. doi: 10.7554/eLife.22502 (PMC5226650; doi:10.7554/eLife.22502)
Supplement: Supplementary file 2. — Only the top 15 combinations are displayed (see Materials and methods, GWAS with pairwise combinations of SNPs). DOI: http://dx.doi.org/10.7554/eLife.22502.019 [file elife-22502-supp2.docx]

**Supplementary file 2. Local association scan using pairwise combination of SNPs.** Only the top 15 combinations are displayed (see Methods, GWAS with pairwise combinations of SNPs).

| **pSNP** | **Rank** | **Chr** | **SNP*i*** | **SNP*j*** | ***P* value** | **Score** |
| --- | --- | --- | --- | --- | --- | --- |
| 285192 | 1 | 5 | 18570773 | 18592365 | 1.87E-12 | 11.73 |
| 579096 | 1 | 5 | 18591361 | 18592365 | 1.87E-12 | 11.73 |
| 580642 | 1 | 5 | 18591702 | 18592365 | 1.87E-12 | 11.73 |
| 581026 | 1 | 5 | 18591703 | 18592365 | 1.87E-12 | 11.73 |
| 285114 | 5 | 5 | 18570773 | 18587728 | 4.18E-12 | 11.38 |
| 285124 | 5 | 5 | 18570773 | 18588230 | 4.18E-12 | 11.38 |
| 572347 | 7 | 5 | 18590327 | 18592365 | 1.38E-11 | 10.86 |
| 285008 | 8 | 5 | 18570773 | 18581639 | 1.50E-11 | 10.83 |
| 285479 | 9 | 5 | 18570773 | 18605986 | 2.24E-11 | 10.65 |
| 532791 | 9 | 5 | 18584397 | 18592365 | 2.25E-11 | 10.65 |
| 396441 | 11 | 5 | 18575807 | 18592365 | 2.86E-11 | 10.54 |
| 480217 | 11 | 5 | 18580387 | 18592365 | 2.86E-11 | 10.54 |
| 285165 | 13 | 5 | 18570773 | 18590369 | 3.77E-11 | 10.42 |
| 285196 | 13 | 5 | 18570773 | 18592705 | 3.77E-11 | 10.42 |
| 285200 | 13 | 5 | 18570773 | 18592873 | 3.77E-11 | 10.42 |
