# Supplementary material for: Multiple alleles at a single locus control seed dormancy in Swedish Arabidopsis
Source: eLife. 2016 Dec 14;5:e22502. doi: 10.7554/eLife.22502 (PMC5226650; doi:10.7554/eLife.22502)
Supplement: Supplementary file 3. — The number of soil samples was adjusted (Soil adj.) to take into account the size of the sampled area and seed transportation over the course of the experiment (see Materials and methods). The mean ﬁtnesses were calculated by dividing the total number of live samples by the number of dispersed viable seeds (Total / Dispersed * Viability). (B) Summary of allele frequency changes at the DOG1 locus, per population and block. (C) Summary of genotype frequency changes at the DOG1 locus, per population and block.(D) Relative ﬁtnesses of DOG1 alleles in ﬁeld experiments, per population and block. DOI: http://dx.doi.org/10.7554/eLife.22502.020 [file elife-22502-supp3.docx]

**Supplementary file 3A. Field experiments summary and sample inventory, per block and population.** The number of soil samples was adjusted (Soil adj.) to take into account the size of the sampled area and seed transportation over the course of the experiment (see Methods). The mean ﬁtnesses were calculated by dividing the total number of live samples by the number of dispersed viable seeds (Total / Dispersed * Viability).

| **Pop.** | **Block** | **Sept. (1)** | **Nov. (2)** | **Soil (3)** | **Soil adj.** | **Total** | **Dispersed** | **Viability** | **Mean ﬁtness** |
| --- | --- | --- | --- | --- | --- | --- | --- | --- | --- |
| 1 | 1 | 76 | 55 | 30 | 1050 | 1181 | 3700 | 0.86 | 0.37 |
| 1 | 2 | 80 | 68 | 32 | 1088 | 1236 | 3700 | 0.86 | 0.39 |
| 2 | 1 | 52 | 39 | 41 | 1394 | 1485 | 3424 | 0.91 | 0.48 |
| 2 | 2 | 69 | 46 | 34 | 1122 | 1237 | 3424 | 0.91 | 0.40 |

**Supplementary file 3B. Summary of allele frequency changes at the *DOG1* locus, per population and block.**

| **Pop.** | **Block** | **Allele** | **Start** | **Sept. (1)** | **Nov. (2)** | **Soil (3)** | **End** |
| --- | --- | --- | --- | --- | --- | --- | --- |
| D2 x ND | 1 | D2 | 0.56 | 0.58 | 0.78 | 0.73 | 0.73 |
| D2 x ND | 1 | ND | 0.44 | 0.42 | 0.22 | 0.27 | 0.27 |
| D2 x ND | 2 | D2 | 0.56 | 0.67 | 0.73 | 0.59 | 0.61 |
| D2 x ND | 2 | ND | 0.44 | 0.33 | 0.27 | 0.41 | 0.39 |
| ND x D3 | 1 | ND | 0.56 | 0.82 | 0.64 | 0.41 | 0.43 |
| ND x D3 | 1 | D3 | 0.44 | 0.18 | 0.36 | 0.59 | 0.57 |
| ND x D3 | 2 | ND | 0.56 | 0.82 | 0.52 | 0.38 | 0.41 |
| ND x D3 | 2 | D3 | 0.44 | 0.18 | 0.48 | 0.62 | 0.59 |

**Supplementary file 3C. Summary of genotype frequency changes at the *DOG1* locus, per population and block.**

| **Pop.** | **Block** | **Genotype** | **Start** | **Sept. (1)** | **Nov. (2)** | **Soil (3)** | **End** |
| --- | --- | --- | --- | --- | --- | --- | --- |
| D2 x ND | 1 | D2/D2 | 0.44 | 0.45 | 0.67 | 0.60 | 0.59 |
| D2 x ND | 1 | D2/ND | 0.24 | 0.26 | 0.22 | 0.27 | 0.26 |
| D2 x ND | 1 | ND/ND | 0.32 | 0.29 | 0.11 | 0.13 | 0.14 |
| D2 x ND | 2 | D2/D2 | 0.44 | 0.55 | 0.59 | 0.47 | 0.48 |
| D2 x ND | 2 | D2/ND | 0.24 | 0.24 | 0.28 | 0.25 | 0.25 |
| D2 x ND | 2 | ND/ND | 0.32 | 0.21 | 0.13 | 0.28 | 0.27 |
| ND x D3 | 1 | ND/ND | 0.45 | 0.69 | 0.53 | 0.27 | 0.29 |
| ND x D3 | 1 | D3/ND | 0.23 | 0.25 | 0.24 | 0.29 | 0.29 |
| ND x D3 | 1 | D3/D3 | 0.32 | 0.06 | 0.24 | 0.44 | 0.42 |
| ND x D3 | 2 | ND/ND | 0.45 | 0.71 | 0.38 | 0.26 | 0.29 |
| ND x D3 | 2 | D3/ND | 0.23 | 0.24 | 0.29 | 0.24 | 0.24 |
| ND x D3 | 2 | D3/D3 | 0.32 | 0.06 | 0.33 | 0.50 | 0.47 |

**Supplementary file 3D. Relative ﬁtnesses of *DOG1* alleles in ﬁeld experiments, per population and block.**

| **Pop.** | **Block** | **D / D** | **D / ND** | **ND / ND** |
| --- | --- | --- | --- | --- |
| D2 x ND | 1 | 1.00 | 0.83 | 0.33 |
| D2 x ND | 2 | 1.00 | 0.97 | 0.77 |
| ND x D3 | 1 | 1.00 | 0.95 | 0.49 |
| ND x D3 | 2 | 1.00 | 0.70 | 0.45 |
